# Frank-Starling and Guyton together at bedside during a fluid challenge

**DOI:** 10.1186/cc10845

**Published:** 2012-03-20

**Authors:** H Aya, M Cecconi, M Geisen, C Ebm, M Grounds, N Fletcher, A Rhodes

**Affiliations:** 1 St George's Hospital, London, UK

## Introduction

According to Guyton, the difference between mean systemic filling pressure (Pms) and right atrial pressure (RAP) is the venous pressure gradient (VP). This is proportional to venous return and cardiac output (CO). According to the Frank-Starling law a fluid challenge successfully increases the stroke volume if the preload increases in the ascending part of the curve. The aim of this study was to assess the significance of the analogue of the Pms (Pmsa) measured with the Navigator™ (Applied Physiology, St Leonards, Australia), the central venous pressure (CVP) (as a surrogate of RAP) and the VP during a fluid challenge.

## Methods

A prospective observational study was performed in postsurgical patients. Patients were monitored with a central venous catheter, an arterial line, a calibrated LiDCO™plus (LiDCO, Cambridge, UK) and the Navigator™. A 250 ml fluid challenge was used to maximise the stroke volume (SV). Data were recorded before and after the fluid challenge which was given over 5 minutes. A positive response to the fluid challenge was defined as either a stroke volume or CO increase of 10% or more.

## Results

Twenty-five fluid challenges in 14 patients were observed. In seven cases (28%), the fluid challenge increased SV (and CO) by ≥10% (Table 1). At baseline there were no differences between HR, Pmsa, CVP or ΔVP for responders or nonresponders. The responders had greater changes in ΔVP in response to the challenge.

**Table 1:** Haemodynamic parameters in responders and nonresponders

	Nonresponders	Responders	*P *value
MAP	78.4	71.4	0.07
Pmsa	17	15	0.3
CVP	9.7	9	0.7
HR	89	91	0.7
ΔVP	7.2	6	0.09
ΔPmsa	19.3	22.1	0.6
ΔCVP	2.9	1.6	0.1
Δ(ΔVP)	1.1	24.8	<0.01

## Conclusion

Our study demonstrates that the Navigator™ may be used to monitor the effect of fluid challenges by assessing the change in VP to the challenge.

